# Quality of antenatal care in 13 sub-Saharan African countries in the SDG era: evidence from Demographic and Health Surveys

**DOI:** 10.1186/s12884-024-06459-2

**Published:** 2024-04-23

**Authors:** Edward Kwabena Ameyaw, Linus Baatiema, Ambrose Naawa, Frederick Odame, Doris Koramah, Francis Arthur-Holmes, Shadrack Osei Frimpong, Celestin Hategeka

**Affiliations:** 1 School of Graduate Studies and Institute of Policy Studies, Lingnan University, Hong Kong, China; 2 L&E Research Consult Ltd, Wa, Upper West Region Ghana; 3 Ghana Health Service, Upper West Regional Health Directorate, Wa, Ghana; 4 Centre for Environment, Migration and International Relations; Faculty of Public Policy and Governance, Simon Diedong Dombo University of Business and Integrated Development Studies, Wa, Ghana; 5 Wits Business School, Faculty of Commerce, Law and Management, University of Witwatersrand, Johannesburg, South Africa; 6 Department of Sociology and Anthropology, College of Humanities and Legal Studies, University of Cape Coast, Cape Coast, Ghana; 7 Department of Sociology and Social Policy, Lingnan University, Hong Kong, China; 8 Yale School of Medicine, Yale University, 333 Cedar St, New Haven, CT 06510 USA; 9 Department of Public Health and Primary Care, University of Cambridge, Cambridge, UK; 10 Boston University, Boston, MA USA

**Keywords:** Antenatal, Quality, Maternal healthcare, Global health, Public health, sub-Saharan Africa

## Abstract

**Background:**

Maternal and neonatal mortality remains high in sub-Saharan Africa (SSA) with women having 1 in 36 lifetime risk. The WHO launched the new comprehensive recommendations/guidelines on antenatal care (ANC) in 2016, which stresses the essence of quality antenatal care. Consequently, the objective of this cross-sectional study is to investigate the quality of ANC in 13 SSA countries.

**Methods:**

This is a cross-sectional study that is premised on pre-existing secondary data, spanning 2015 to 2021. Data for the study was obtained from the Measure DHS Programme and included a total of 79,725 women aged 15–49 were included. The outcome variable was quality ANC and it was derived as a composite variable from four main ANC services: blood pressure taken, urine taken, receipt of iron supplementation and blood sample taken. Thirteen independent variables were included and broadly categorised into individual and community-level characteristics. Descriptive statistics were used to present the proportion of women who had quality ANC across the respective countries. A two-level multilevel regression analysis was conducted to ascertain the direction of association between quality ANC and the independent variables.

**Results:**

The overall average of women who had quality ANC was 53.8% [CI = 51.2,57.5] spanning from 82.3% [CI = 80.6,85.3] in Cameroon to 11% [CI = 10.0, 11.4] in Burundi. Women with secondary/higher education had higher odds of obtaining quality ANC compared with those without formal education [aOR = 1.23, Credible Interval [Crl] = 1.10,1.37]. Poorest women were more likely to have quality ANC relative to the richest women [aOR = 1.21, Crl = 1.14,1.27]. Married women were more likely to receive quality ANC relative to those cohabiting [aOR = 2.04, Crl = 1.94,3.05]. Women who had four or more ANC visits had higher odds of quality ANC [aOR = 2.21, Crl = 2.04,2.38]. Variation existed in receipt of quality ANC at the community-level [σ^2^ = 0.29, Crl = 0.24,0.33]. The findings also indicated that a 36.2% variation in quality ANC is attributable to community-level factors.

**Conclusion:**

To achieve significant improvement in the coverage of quality ANC, the focus of maternal health interventions ought to prioritise uneducated women, those cohabiting, and those who are unable to have at least four ANCs. Further, ample recognition should be accorded to the existing and potential facilitators and barriers to quality ANC across and within countries.

## Background

Maternal and neonatal mortality remains high in sub-Saharan Africa (SSA) with women having a 1 in 36 lifetime risk of maternal mortality [1]. Ensuring reduction in maternal ill health and adverse health outcomes is highly prioritised by the United Nations’ Global Strategy for Women’s, Children’s, and Adolescent Health (2016–2030) [2] and the Sustainable Development Goals (SDGs) [3]. Targets one and two of the third SDG require that maternal mortality be reduced to 70 maternal deaths per 100,000 live births and neonatal deaths to 12 deaths per 1,000 live births by 2030 [3]. Globally, about 210 million pregnancies occur every year and adverse consequences of these pregnancies can be mitigated with quality antenatal care (ANC) [4, 5].

To safeguard the well-being of all pregnant women and their foetuses, the WHO launched new comprehensive recommendations/guidelines on ANC in 2016 [6–8]. However, recent evidence has shown that while coverage of maternal and child health services has increased during the MDG era, quality still lagged [9, 10]. Quality in ANC is assessed by the number of recommended ANC services a woman receives during pregnancy, including blood pressure checks, taking the woman’s urine and blood samples as well as the receipt of iron supplement [6]. Hence, receipt of these essential services is a proxy for quality ANC service. Bolstering health outcomes requires optimizing both coverage and quality of services [10]. WHO highlights the essence of quality ANC, thus the content or specific services provided to women during ANC visits. The frequency of ANC visits is, however, deficient in providing information about the content or component of services received.

As a result, the content of care received during ANC, termed effective, has been adjudged as an indispensable dimension for ANC indicator development [11]. Thus, scholars posit that effective coverage is essential for measuring the quality of ANC [12, 13]. Besides, the WHO guidelines stress the content/components of high-quality ANC at each visit. However, the quality of ANC, being the receipt of essential ANC services, has received limited research attention in SSA, where the majority of maternity ill-health conditions occur. Previous studies on ANC have predominantly focused on ANC attendance and its correlates [14–16]. The few available studies on quality ANC in SSA explored the phenomenon from a care provision perspective, thus the readiness of facilities to provide the recommended ANC services [17, 18] without inquiring from the women if such services were received. This study addresses the existing literature gap by using nationally representative comparable cross-sectional surveys to investigate the quality of ANC in SSA. Outcome of the study may help inform policy decisions including improvement strategies targeting maternal and newborn health across the region and enhance prospects of achieving maternal and newborn health-related SDG targets.

## Methods

### Study design and data sources

This is a cross-sectional study based on pre-existing secondary data from the Demographic and Health Survey. The dataset spanned 2015–2021. Demographic and Health Surveys (DHSs) are nationally representative cross-sectional population-based surveys carried out in multiple low and middle-income countries. They usually include 5,000 to 30,000 households [19]. DHS data are gathered with a standard model questionnaire, however, the questionnaire may be adapted and it is permissible to include optional modules. The data are usually based on self-reports from women. Data on ANC are obtained for the recent pregnancy that resulted in a live birth within a defined recall period, which is usually within five years [19]. The sampling design usually follows a multilevel cluster survey approach [20]. The target for the survey includes all women aged 15 and 49 [19]. The present study comprised 79,725 women from thirteen countries in SSA.

### Outcome variable derivation

Quality ANC was the outcome of interest in this study. This was gauged as the proportion of women who reported that they received four essential services during any ANC, namely: blood pressure taken (1), urine taken (2), iron supplement given (3) and; blood sample taken (4). These four services were aggregated such that women who received all the services were considered to have obtained quality ANC (coded 1), whilst any woman who did not receive all these four services was categorised otherwise (coded 0). For quality ANC, a woman should receive more than these four services [2], however, four services were used in the present study due to variables’ availability, comparability and survey wave of DHS across SSA. We omitted IPTp because the missing responses for this variable were significantly high in some of the countries.

### Independent variables’ derivation

**Fig. 1:**
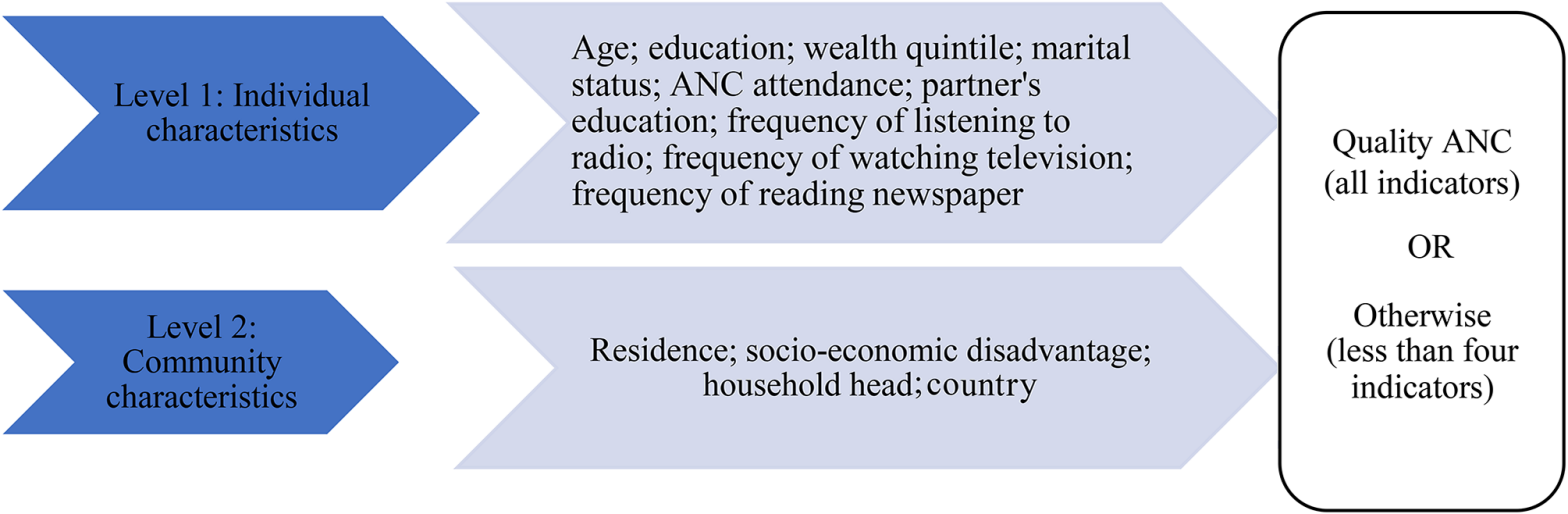
Conceptual model of relationship between covariates and quality ANC

Based on existing literature [5, 7, 8, 12, 16, 21, 22] we included thirteen independent variables hierarchically layered into two levels: individual and community-level characteristics (see Fig. 1). The individual-level variables were *age* (categorized into 15–19, 20–24, 25–29, 30–34. 35–39, 40–44, 45–49); *education* (coded as no education, primary, secondary/higher); *wealth* (categorised into poorest, poorer, middle, richer, richest); *marital status* (coded as married, cohabiting and single); *ANC attendance* (coded into less than four (< 4) and four or more (≥ 4)). *Partner’s education* was categorised into no education, primary and secondary/higher. Three of the variables (frequency of listening to radio, frequency of watching television and frequency of reading newspaper) were coded as not at all, less than once a week, at least once a week; and almost every day. At the community level, *residence* (rural/urban), *socio-economic disadvantage* (tertile 1, 2, 3), *household head* (male or female) and *country* (Burundi, Malawi, Uganda, Chad, Tanzania, Zambia, Angola, Senegal, Mali, Guinea, Nigeria, Benin, Cameroon) were fitted.

### Analytical strategy

Descriptive statistics were used to present the proportion of women who had quality ANC in the included countries. In addition, the proportion of women who had quality ANC was computed by individual, and community-level factors and chi-square associations were explored. Further, multilevel regression analyses at two levels were conducted to ascertain the direction of association between quality ANC and the independent (individual and community level) variables, thereby resulting in five models.

In the first model, no independent variable was included (Model I). This model was unconditional and aided in examining the magnitude of variance between individual and community levels. The second model accounted for individual-level variables and quality ANC (Model II). The third model included community-level variables and quality ANC (Model III). In the final model IV- all the independent variables were included to ascertain how they jointly interact to inform quality ANC in SSA. The analyses generated two kinds of results; fixed effects and random effects. The fixed effects were composed of adjusted odds ratios at 95% credible intervals. In the case of random effects, the results were presented as variance partition coefficient (VPC) and median odds ratio (MOR) [23]. The VPC estimates the magnitude of variance in the likelihood of quality ANC that is attributable to community-level factors. MOR quantified community variance concerning odds ratios and also estimated the prospects of quality ANC that is affected by community-level factors. All analyses were done using Stata version 13.

### Model fit and specifications

Multicollinearity was assessed with the Variance Inflation Factor (VIF) [24]. The results indicated that none of the explanatory variables were highly correlated (mean VIF = 2.53, minimum VIF = 1.55, maximum VIF = 3.98). We used the Bayesian Deviance Information Criterion (DIC) to assess the goodness of fit of the models. Additionally, Markov Chain Monte Carlo (MCMC) estimation was used in the multilevel logistic regression modelling [25]. Statistical significance was fixed at 95% confidence interval and modelling operations were executed using 3.05 version of MLwinN package.

### Ethical approval

Since secondary data was used, ethical approval for this type of study is not required by our institute. For all surveys included, the DHS Program sought ethical approval from the Ethics Committee of ORC Macro Inc. Additional ethical clearance was obtained from the Ethics Boards of all partner organisations of the included countries. Written or verbal consent was obtained from each participant. For the current study, we did not seek further ethical approval as the data is freely available to the public. We accessed the data by placing a request with the DHS Program. Detailed information about how to access the DHS data and ethical standards can be accessed here: available at http://goo.gl/ny8T6X.

## Results

### Socio-demographic characteristics by quality ANC

A little above half of women aged 25–29 had quality ANC (52.9%) (see Table 1). Quality ANC dominated among women with secondary/higher education (66.2%) and richest women (65.7%). At least five out of ten married (53.5%) and single (51.2%) women had quality ANC. Seven out of ten women who obtained four or more ANC visits had quality ANC (71.0%), meanwhile approximately half of those with less than four ANC visits received quality ANC (49.3%). Similarly, a greater proportion of women whose partners had secondary/higher education obtained quality ANC (62.6%). Most women who listened to radio almost every day (66.7), watched television almost every day (78.2%) or read the newspaper almost every day (69.9%) received quality ANC. The proportion of urban residents who had quality ANC (70.0%) exceeded the rural women who had quality ANC (43.8%). Only about half of the least socio-economically disadvantaged women received quality ANC (53.6%). Also, there was a slight disparity in the proportion of women from male (51.3%) and female-headed (50.9%) households who had quality ANC.

**Table 1:** Socio-demographic characteristics by quality ANC (*n* = 79,725)

Variable	Quality ANC		Total
	Yes	No	
	n (%, row)	n (%, row)	n (%, row)
**Individual level**			
Age			
15–19	2,300(47.1)	2,582(52.9)	4,882(100)
20–24	8,553(49.1)	8,874(50.9)	17,427(100)
25–29	10,854(52.9)	9,660(47.1)	20,514(100)
30–34	8,751(52.1)	8,058(47.9)	16,809(100)
35–39	6,322(52.1)	5,806(47.9)	12,128(100)
40–44	3,016(51.0)	2,900(49.0)	5,916(100)
45–49	1,047(51.2)	1,002(48.9)	2,049(100)
Education			
No education	16,444(52.5)	14,896(47.5)	31,340(100)
Primary	11,485(39.8)	17,407(60.2)	28,892(100)
Secondary/higher	12,914(66.2)	6,579(33.8)	19,493(100)
Wealth			
Poorest	6,488(39.8)	9,831(60.2)	16,319(100)
Poorer	7,524(44.8)	9,290(55.3)	16,814(100)
Middle	8,262(50.5)	8,097(49.5)	16,359(100)
Richer	8,894(57.3)	6,618(42.7)	15,512(100)
Richest	9,675(65.7)	5,046(34.3)	14,721(100)
Marital status			
Married	36,622(53.5)	31,892(46.5)	68,514(100)
Cohabiting	3,882(36.7)	6,691(63.3)	10,573(100)
Single	339(51.2)	299(46.8)	638(100)
ANC attendance			
< 4	23,091(49.3)	24,034(50.7)	47,125(100)
≥ 4	23,146(71.0)	9,454(29.0)	32,600(100)
Partner’s education			
No education	14,185(52.9)	12,619(47.1)	26,804(100)
Primary	9,857(37.8)	16,202(62.2)	26,059(100)
Secondary/higher	16,801(62.6)	10,061(37.4)	26,862(100)
Frequency of listening to radio			
Not at all	14,749(44.9)	18,105(55.1)	32,854(100)
Less than once a week	9,106(54.6)	7,558(45.4)	16,664(100)
At least once a week	16,347(55.9)	12,899(44.1)	29,246(100)
Almost every day	641(66.7)	320(33.3)	961(100)
Frequency of watching television			
Not at all	21,508(41.6)	30,126(58.4)	51,634(100)
Less than once a week	6,671(62.5)	4,006(37.5)	10,677(100)
At least once a week	11,789(72.3)	4,506(27.7)	16,295(100)
Almost every day	875(78.2)	244(21.8)	1,119(100)
Frequency of reading newspaper			
Not at all	34,409(49.7)	34,827(50.3)	69,236(100)
Less than once a week	3,971(58.8)	2,783(41.2)	6,754(100)
At least once a week	2,398(65.8)	1,244(34.2)	3,642(100)
Almost every day	65(69.9)	28(30.1)	93(100)
**Community level factors**			
Residence			
Urban	15,813(70.0)	6,790(30.0)	22,603(100)
Rural	25,030(43.8)	32,092(56.2)	57,122(100)
Socio-economic disadvantage			
Tertile 1 (least disadvantaged)	14,284(53.6)	12,348(46.4)	26,632(100)
Tertile 2	13,252(49.9)	13,276(50.1)	26,528(100)
Tertile 3 (most disadvantaged)	13,307(50.1)	13,258(49.9)	26,565(100)
Household head			
Male	35,593(51.3)	33,827(48.7)	69,420(100)
Female	5,250(50.9)	5,055(49.1)	10,305(100)
Total	40,843(51.2)	38,882(48.8)	79,725(100)

χ^2^ = Chi-square, df = degree of freedom

### Proportion of quality of ANC

**Fig. 2:**
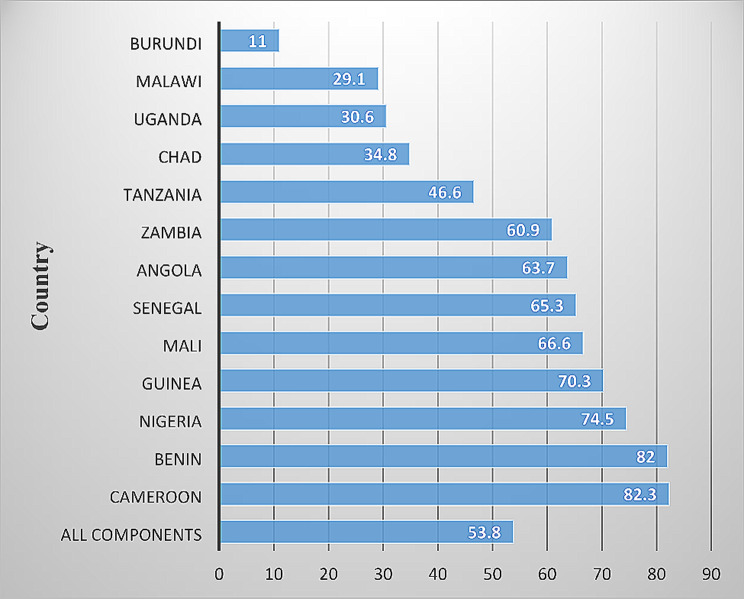
Quality ANC per country

As shown in Fig. 2, the overall percentage of women who had quality ANC was 53.8%. At least eight out of ten women in Cameroon had quality ANC (82.3%; CI = 80.6, 85.3). Meanwhile, a few women in Burundi received quality ANC (11.0%; CI = 10.0, 11.4).

### Predictors of quality ANC

#### Fixed effects

From Model V, women with secondary/higher education had higher odds of obtaining quality ANC compared with those without formal education [aOR = 1.23, Crl = 1.10,1.37] (see Table 2). Poorest women were more likely to have quality ANC relative to richest women [aOR = 1.21, Crl = 1.14,1.27]. Married women were more likely to receive quality ANC relative to those cohabiting [aOR = 2.04, Crl = 1.94,3.05]. Women who had four or more ANC visits had higher odds of quality ANC [aOR = 2.21, Crl = 2.04,2.38]. Relative to women whose partners had no formal education, those whose partners had secondary/higher education were more likely to have received quality ANC [aOR = 1.14, CrI = 1.09,1.18]. Women who listened to radio almost every day had higher odds of quality ANC [aOR = 1.61, Crl = 1.37,1.89]. Similarly, those who watched television almost every [aOR = 2.53, Crl = 2.12,2.97] and those who read a newspaper at least once a week had higher odds of attaining quality ANC [aOR = 1.14, Crl = 1.05,1.26]. Compared to urban women, rural women had lower odds of quality ANC [aOR = 0.49, Crl = 0.48,0.80]. Relative to Cameroonian women, women of all other nationalities with significant association had lower odds of obtaining quality ANC especially Chad [aOR = 0.13, Crl = 0.09–0.92].

**Table 2:** Multilevel regression results

Variable	Model I	Model II		Model III		Model IV	
	Empty Model	aOR	[95% Crl]	aOR	[95% Crl]	aOR	[95% Crl]
Fixed effects							
**Individual-level factors**							
Age							
15–19		0.99	[0.93,1.07]			1.00	[0.95,1.07]
20–24		Ref.	Ref.			Ref.	Ref.
25–29		1.04	[0.99,1.08]			1.04	[0.97,1.08]
30–34		0.98	[0.94,1.03]			0.98	[0.94,1.03]
35–39		1.01	[0.96,1.07]			1.01	[0.97,1.06]
40–44		1.03	[0.97,1.10]			1.05	[0.96,1.11]
45–49		1.06	[0.96,1.17]			1.08	[0.97,1.17]
Education							
No education		Ref.	Ref.			Ref.	Ref.
Primary		0.65^***^	[0.63,0.68]			0.66^***^	[0.62,0.69]
Secondary/higher		1.22^***^	[1.10,1.35]			1.23^***^	[1.10,1.37]
Wealth							
Poorest		0.79^***^	[0.74,0.84]			1.21^***^	[1.14,1.27]
Poorer		0.91^***^	[0.86,0.96]			1.48^***^	[1.30,1.49]
Middle		0.99	[0.94,1.06]			1.44^***^	[1.36,1.52]
Richer		1.09^**^	[1.03,1.15]			1.38^***^	[1.31,1.46]
Richest		Ref.	Ref.			Ref.	Ref.
Marital status							
Married		1.66^***^	[1.59,1.73]			2.04^***^	[1.94,3.05]
Cohabiting		Ref.	Ref.			Ref.	Ref.
Single		1.40^***^	[1.18,1.66]			1.81^***^	[1.29,2.93]
ANC attendance							
< 4		Ref.	Ref.			Ref.	Ref.
≥ 4		2.33^***^	[2.17,2.50]			2.21^***^	[2.04,2.38]
Partner’s education							
No education		Ref.	Ref.			Ref.	Ref.
Primary		0.67^***^	[0.64,0.70]			0.69^***^	[0.67,0.70]
Secondary/higher		1.11^***^	[1.06,1.17]			1.14^***^	[1.09,1.18]
Frequency of listening to radio							
Not at all		Ref.	Ref.			Ref.	Ref.
Less than once a week		1.07^**^	[1.06,1.17]			1.08^**^	[1.03,1.13]
At least once a week		1.01	[0.98,1.05]			1.05^*^	[1.02,1.09]
Almost every day		1.59^***^	[1.35,1.86]			1.61^***^	[1.37,1.89]
Frequency of watching television							
Not at all		Ref.	Ref.			Ref.	Ref.
Less than once a week		1.92^***^	[1.84,2.02]			1.82^***^	[1.74,1.91]
At least once a week		2.55^***^	[2.43,2.68]			2.26^***^	[2.19,2.39]
Almost every day		3.09^***^	[2.62,3.65]			2.53^***^	[2.12,2.97]
Frequency of reading newspaper							
Not at all		Ref.	Ref.			Ref.	Ref.
Less than once a week		1.01	[0.96,1.07]			1.03	[0.97,1.09]
At least once a week		1.12^*^	[1.02,1.21]			1.14^**^	[1.05,1.26]
Almost every day		1.06	[0.64,1.76]			1.08	[0.65,1.79]
**Community level factors**							
Residence							
Urban				Ref.	Ref.	Ref.	Ref.
Rural				0.32^***^	[0.31,0.33]	0.49^***^	[0.48,0.80]
Socio-economic disadvantage							
Tertile 1 (least disadvantaged)				Ref.	Ref.	Ref.	Ref.
Tertile 2				0.80^***^	[0.74,0.86]	1.06	[0.99,1.14]
Tertile 3 (most disadvantaged)				0.83^***^	[0.76,0.90]	1.06	[0.99,1.14]
Country							
Burundi						Ref.	Ref.
Malawi						1.28	[0.99,3.11]
Uganda						0.55***	[0.33,0.89]
Chad						0.13**	[0.09,0.92]
Tanzania						0.69	[0.40,1.51]
Zambia						0.29***	[0.13,0.40]
Angola						0.82	[0.70,1.65]
Senegal						0.47	[0.27,1.54]
Mali						0.80*	[0.65,0.87]
Guinea						0.56	[0.11,1.33]
Nigeria						0.71**	[0.50,0.87]
Benin						0.60*	[0.22,0.94]
Cameroon						1.19**	[1.04,2.03]
**Random-effect**							
Community-level							
Variance [95% CrI]	0.29[0.24,0.33]	0.13	[0.11,0.16]	0.22	[0.18,0.25]	0.15	[0.10,0.17]
VPC % [95% Crl]	36.2[25.9,48.1]	33.0	[24.4,51.5]	34.2	[25.0,44.8]	35.0	[22.8,52.9]
MOR [95% CrI]	1.67[1.60,1.73]	1.41	[1.37,2.79]	1.57	[1.50,1.60]	1.44	[1.40,1.49]
Explained variation (%)	Ref.	55.2		25.8		52.1	
**Model fit statistics**							
Bayesian DIC	108,124	99,663		104,007		99,390	
**Sample size**							
Community-level	1,395	1,395		1,395		1,395	
Individual level	79,725	79,725		79,725		79,725	

aOR = adjusted Odds Ratio; CrI = Credible Interval; VPC = Variance Partition Coefficient; MOR = Median Odds Ratio

1 = reference; ^*^p < 0.05, ^**^p < 0.01, ^***^p < 0.001

### Random-effects

We presented the results of the random effects in Table 2. The empty model (Model I) revealed that variation exists in receipt of quality ANC at the community [σ^2^ = 0.29, Crl = 0.24,0.33] level. Through the VPCs, the same model indicated that 36.2% variation in quality ANC is attributable to community-level factors. The MOR of the final model (Model IV**)** showed that the chances of a woman receiving quality ANC when she relocates to a different community with high chance of quality ANC is 44% chance of receiving quality ANC.

## Discussion

We analysed recent DHS data of thirteen SSA countries to investigate quality ANC. Quality ANC was ranged from 82.3% in Cameroon to 11% in Burundi, averaging 53.8%. Women with secondary/higher education had higher odds of obtaining quality ANC compared with those without formal education. Poorest women were more likely to have quality ANC relative to the richest women. Married women were more likely to receive quality ANC relative to those cohabiting. Women who had four or more ANC visits had higher odds of quality ANC. Variation existed in receipt of quality ANC at the community level with 36.2% variation in quality ANC being attributable to community-level factors.

Compared to Cameroon women, women of all other nationalities had lower odds of quality ANC especially Chad. This depicts an inter-country variability in ANC quality. This may reflect how maternity healthcare is structured across sub-Saharan African countries, variations in governments’ commitment and women’s acceptability/utilisation of available maternity care. A recent study from Chad revealed that maternal healthcare utilization tends to be generally low (7%). This may reduce the chances of obtaining quality ANC hence our finding [26]. Besides, access to healthcare is a major problem in Chad [27]. However, there are ongoing maternal health initiatives such as the Chad Mother and Child Health Services Strengthening Project [28]. As a result, it is possible that the impact of ongoing interventions are yet to manifest in the area of quality ANC. On the other hand, it can be conjectured that ongoing interventions in Cameroon are relatively effective in ensuring that women receive quality ANC. Since 2011, there has been a conscious and synergistic effort between the Cameroonian government, the World Bank and other partner organisations to enhance maternity care. This manifests in the program called Performance-Based Financing (PBF) [29]. These and other factors may be the factors leading to the observation made about Cameroon.

Women with secondary/higher education had higher odds of quality ANC compared with those without formal education. This finding concurs with the reported positive association between maternal education and utilization of maternal healthcare services [21, 30–32]. Similarly, women who listened to radio almost every day had higher odds of quality ANC. Those who watched television almost every day and those who read a newspaper at least once a week also had an increased likelihood of quality ANC. To a greater extent, women with high media exposure (radio/TV) are likely to be educated, hence the findings are anticipated. Education enhances quality ANC through several pathways. First, with education, women are enlightened about the benefits of getting blood pressure taken, blood sample and urine taken. Consequently, educated women may be more knowledgeable about the benefits and are more likely to insist and ensure that they receive quality ANC [33–35].

Second, education being an indicator of empowerment may boost women’s negotiation skills and confidence to ask ANC providers to offer them the core services they need during each trimester [34]. However, it will be prudent for health professionals to offer quality ANC to everyone regardless of their educational attainment in order to partly address some of the critical dimensions of vulnerability to poor quality of care [10]. The finding underscores the essence of governments of included countries to enhance females’ prospects of formal education. This is urgently required on the account that education has enormous implications on the quality of ANC, which in turn affects pregnancy outcomes as reported from low and middle-income countries [7, 36]. Generally, mass media has been acknowledged as efficacious in enhancing maternal healthcare utilization [37]. This is because radio and television stations usually communicate in the local language(s) within their jurisdiction of operation. The media, especially radio seem to have a very wide coverage in SSA [38]. Subsequently, realizing that women with high exposure to radio and television highlight that the mass media can be utilized effectively to ensure that women receive the core components of ANC [39–41].

Relative to women whose partners had no formal education, those whose partners had secondary/higher education were more likely to have quality ANC. In SSA, households are usually headed by men [42, 43]. Educational status and depth of knowledge of these men would eventually influence their choices and decisions and affect their wives and households [14]. As a result, educated men, being literate can easily read and appreciate the need for women to receive the requisite components of ANC [39, 44, 45]. These men can easily accompany their wives during ANC visits and investigate to make sure that their wives receive all the required services and components of care. Since formal education is not the only means of knowledge acquisition, health sectors of the included countries can tailor ANC advocacy campaigns targeting women whose partners have no formal education. This may encourage women whose partners have no formal education to appreciate the need to frequent ANC and obtain all required services/components.

The findings showed that the poorest women had higher odds of quality ANC relative to richest women. This is inconsistent with the literature due to the cost of healthcare and high possibility for the poor to stay in less advantaged neighborhoods and distant locations from health facilities [46, 47]. These notwithstanding, our finding is plausible due to ongoing pro-poor interventions by SSA governments aimed at ensuring universal health coverage (UHC). One of such interventions is the edge-cutting health insurance scheme, which operates in several countries across SSA [48]. It is however noteworthy that health insurance schemes across SSA are marked with some notable nuances concerning the target population, mode of premium payment and extent of coverage. For instance, although health insurance is operational in Ghana, Kenya, Tanzania and Zimbabwe; notable variations exist [49–52]. In spite of these, the ultimate goal of these insurance schemes is to bridge the health inequity gap between the poor and the rich. Consequently, it is plausible that a substantial proportion of poor women who participated in the surveys were subscribed to health insurance and making good use of it. Further, it is well established in the literature that women who are subscribed to health insurance have high maternal healthcare utilization [53–55].

Married women were more likely to have quality ANC relative to those cohabiting. Unlike other women, those married may have support from their husbands in the form of reminders and accompaniment to ANC [38]. In addition, a married woman would likely attend ANC clinic and can confidently ask healthcare providers for all essential services with ease due to societal acceptance of pregnancies within marriage [56]. This may not be the situation for a woman who is cohabiting because cohabitation is labelled as illegitimate in most SSA societies [57]. Consequently, pregnant women who are cohabiting have higher chances of not meeting the required ANC services as some of them may be less motivated to access healthcare whilst bearing out-of-wedlock pregnancies, fear of mockery or fear of having a higher chance of being scolded by the health care providers.

Women who had four or more ANC visits had higher odds of quality ANC. The enormous benefits of ANC on maternal and newborn health outcomes cannot be overemphasized [2, 58]. Through ANC, healthcare professionals can educate women about maternity best practices and administer all essential medications [2]. On this premise, it is anticipated that women with high ANC attendance would receive the full content of ANC from the first to third trimester. This finding is suggestive of the need to initiate frequent reminders and encourage women to achieve the recommended ANC visits. Context-specific media and local engagements may be utilized in targeting women for this course.

Rural women had lower odds of quality ANC. Several factors dissuade rural residents from obtaining the essential components of ANC [59]. Across SSA, a plethora of evidence has revealed that health facilities are disproportionate to the detriment of rural residents [22, 60, 61]. Further, some healthcare providers refuse postings to rural settings [62, 63]. As espoused by the three-delays model, travelling long distances to access healthcare causes a second delay and can increase the chances of adverse maternal health outcomes [64]. These and several other factors such as the absence of essential equipment and the reluctance of health personnel to work in rural settings account for rural-urban disparity in quality ANC [65–67]. It is time for governments of included countries to re-assess drivers of health facility allocation and distribution of health personnel.

Considering the foregoing discussion, our finding on variation in quality ANC at community-level is anticipated. The VPCs further demonstrated that community-level variations are also significant indications of quality ANC. These findings illustrate the need for stakeholders in maternal health, particularly SSA governments and their partners to desist from generalized interventions and rather focus on context (community) responsive and relevant measures that can enhance the current status quo of ANC quality.

## Strengths and limitations

The study has some compelling strengths that are worthy of acknowledgment. First is the utilisation of large representative sample from the included countries, which renders the findings and recommendations generalizable to reproductive-aged women in the countries studied. Second, the rigorous analytical procedure helped to generate robust and reliable findings. The limitations include the possibility of recall and social desirability biases as our study utilized self-reported data. The cross-sectional study design does not permit causal inference between the explanatory variables and quality ANC. Also, quality ANC was limited to four contents of ANC due to limited ANC indicators in the DHS and the different indicators collected by various countries.

## Conclusion

A little over half of women in SSA obtained quality ANC. In addition, individual-level factors and community-level issues affect women’s prospects of receiving quality ANC. The study highlights that to achieve significant improvement in the coverage of quality ANC, the focus of maternal health interventions ought to prioritise uneducated women, women who cohabit, those who are unable to have at least four ANCs as well as women with limited or no encounter with the media (radio, television and newspaper). To achieve this, ample recognition should be accorded to the existing and potential facilitators and barriers to quality ANC across and within countries. Qualitative inquiry on facilitators and barriers to quality ANC from health care providers and service users (women’s) perspective is vital and may present a balanced and detailed account to further inform maternal health policies and thereby facilitate the prospects of achieving SDG targets 3.1 and 3.2 in SSA [3].

## Data Availability

Data used for the study is freely available to the public and available at http://goo.gl/ny8T6X.

## Abbreviations

ANC: Antenatal Care
aOR: Adjusted Odds Ratio
CI: Confidence Interval
Crl: Credible Interval
DHS: Demographic and Health Survey
MCMC: Markov Chain Monte Carlo
MDG: Millennium Development Goal
MOR: Median Odds Ratio
PBF: Performance-Based Financing
SDG: Sustainable Development Goal
SSA: sub-Saharan Africa
VIF: Variance Inflation Factor
VPC: Variance Partition Coefficient
WHO: World Health Organisation
